# Assessing Renoprotective Effects of Empagliflozin and Telmisartan Combination Therapy in Non-albuminuric Diabetic Nephropathy: A Retrospective Cohort Study

**DOI:** 10.7759/cureus.100420

**Published:** 2025-12-30

**Authors:** Anand Acharya, Chandra Mouli Krishna Kotakala, Burli Kodanda Ramu, Tanvi Padala, Palle Aditya Reddy, Prashanth Kumar Patnaik, Blessy Niharika Mede

**Affiliations:** 1 Department of Pharmacology, Konaseema Institute of Medical Sciences and Research Foundation, Amalapuram, IND; 2 Department of Pharmacology, Maharajah's Institute of Medical Sciences, Vizianagaram, IND; 3 Department of Internal Medicine, RVM Institute of Medical Sciences and Research Center, Laxmakkapally, IND; 4 Department of Pharmacology, RVM Institute of Medical Sciences and Research Center, Laxmakkapally, IND; 5 Department of Pathology, Mahavir Institute of Medical Sciences, Vikarabad, IND

**Keywords:** egfr slope, normoalbuminuric dkd, renin-angiotensin system blockade, sglt2 inhibitor, urine albumin-to-creatinine ratio

## Abstract

Background: Non-albuminuric diabetic kidney disease (DKD) can exhibit “silent” loss of estimated glomerular filtration rate (eGFR) despite normal urine albumin. We examined whether sodium-glucose cotransporter 2 (SGLT2) inhibition added to angiotensin receptor blockade is associated with slower eGFR decline in this phenotype.

Methods: In this retrospective cohort, adults with type 2 diabetes and a non-albuminuric DKD phenotype (urine albumin-to-creatinine ratio (UACR) <30 mg/g on serial testing, with prespecified evidence supporting DKD when baseline eGFR was 60-89 mL/min/1.73 m^2^) were grouped as concurrent empagliflozin plus telmisartan (n=60) or telmisartan-based care without any SGLT2 inhibitor (n=60). The primary outcome was annualized eGFR slope estimated from repeated measures using weighted linear mixed-effects models.

Results: The analytic cohort included 120 adults with non-albuminuric DKD (empagliflozin + telmisartan, n=60; telmisartan without any SGLT2 inhibitor, n=60). After inverse probability of treatment weighting (IPTW), covariate balance was acceptable (all standardized mean differences <0.10). In weighted linear mixed-effects models, the annualized eGFR slope (negative values indicate decline) was -1.15 mL/min/1.73 m^2^/year in the empagliflozin + telmisartan group and -2.18 mL/min/1.73 m^2^/year in the telmisartan-only group, yielding a between-group difference of +1.03 mL/min/1.73 m^2^/year (slower decline with combination therapy). For event-based outcomes, renal progression occurred in 3/60 (5.0%) with empagliflozin + telmisartan versus 6/60 (10.0%) with telmisartan-only (hazard ratio (HR) 0.50, 95% confidence interval (CI) 0.12-2.06). Incident albuminuria occurred in 5/60 (8.3%) versus 8/60 (13.3%), respectively (odds ratio (OR) 0.59, 95% CI 0.18-1.88).

Conclusions: Combination therapy was associated with slower longitudinal eGFR loss, while event-based outcomes were imprecise, supporting larger prospective validation in normoalbuminuric DKD.

## Introduction

Non-albuminuric diabetic kidney disease (DKD) is increasingly recognised as a clinically important phenotype in which glomerular filtration can decline despite urinary albumin remaining in the normal range, complicating early risk recognition and timely intensification of renoprotective therapy [1,2]. While proteinuria is a potent driver of progressive nephron injury, its absence does not imply biological quiescence; tubulointerstitial inflammation, endothelial dysfunction, hypoxia, and fibrosis may still accumulate and translate into measurable estimated glomerular filtration rate (eGFR) loss [1,3]. In real-world settings, longitudinal cohorts of normoalbuminuric DKD have demonstrated clinically meaningful eGFR decline trajectories, reinforcing that “silent” progression is plausible and warrants proactive disease-modifying strategies [4].

Mechanistically, combining maximally tolerated renin-angiotensin system (RAS) blockade with a sodium-glucose cotransporter 2 (SGLT2) inhibitor is biologically attractive because the two classes target overlapping yet distinct pathways that converge on hyperfiltration, oxidative stress-inflammation, and fibrotic remodeling [5-7]. Angiotensin receptor blockers (ARBs), such as telmisartan, mitigate angiotensin II-mediated intraglomerular hypertension and profibrotic signaling, forming a long-standing cornerstone of nephroprotection. Meanwhile, SGLT2 inhibition exerts hemodynamic and pleiotropic effects that extend beyond glucose lowering [5,8]. Preclinical work further supports convergent anti-fibrotic signatures, with empagliflozin demonstrating fibrosis reduction comparable to telmisartan in chronic kidney disease (CKD) models, though context-dependent neutral findings have also been reported, underscoring the need for phenotype-specific clinical validation [9-11].

However, most clinical evidence and therapeutic algorithms have been anchored to albuminuric risk strata, leaving the non-albuminuric DKD subgroup comparatively understudied [12,13]. In one normoalbuminuric DKD retrospective cohort, SGLT2 inhibitor use was associated with a slower annual eGFR decline than non-use (-1.15 vs. -2.18 mL/min/1.73 m^2^/year), with an apparent amplification among those receiving concomitant RAS inhibition [4]. Building on this gap, we evaluated whether empagliflozin-telmisartan combination therapy is associated with slower kidney function decline compared with a comparator regimen in non-albuminuric DKD, structured to foreground phenotype, mechanistic rationale, and the study objective.

## Materials and methods

Study design, setting, and timeline

This record-based retrospective cohort study evaluated whether empagliflozin plus telmisartan is associated with a different longitudinal kidney function trajectory than telmisartan-based care without SGLT2 inhibitor therapy among adults with type 2 diabetes (T2D) and non-albuminuric DKD. The study was conducted at Konaseema Institute of Medical Sciences and Research Foundation (KIMS & RF), Amalapuram, Andhra Pradesh, India, using routinely collected clinical and laboratory data from institutional health information systems. The study was initiated after Institutional Ethics Committee (IEC) approval (IEC/CD/2024; 24 February 2024), and all data handling followed IEC requirements for retrospective record review.

The retrospective observation window was 01 January 2019 to 31 December 2023. Follow-up began at each patient’s index date and continued until the earliest of last available creatinine/eGFR measurement, death, renal replacement therapy initiation, or study end. Outcome ascertainment was permitted through 30 June 2024 to capture delayed laboratory measurements.

Participants

Because this was a retrospective cohort, the final sample size was determined by the number of eligible patients in the database during the prespecified period. Sixty eligible samples were sorted into each group based on the inclusion and exclusion criteria.

Eligible patients were adults (≥18 years) with confirmed T2D and a non-albuminuric DKD phenotype. DKD was operationally defined using a hierarchy to reduce misclassification: (i) eGFR <60 mL/min/1.73 m^2^ on ≥2 measurements separated by ≥90 days (CKD chronicity), or (ii) eGFR 60-89 mL/min/1.73 m^2^ with clinician-documented DKD/diabetic nephropathy in nephrology/diabetology records plus at least one supportive feature, including documented diabetic retinopathy, a persistent decline in eGFR over three months or longer on serial testing, or renal ultrasound/clinical documentation of chronic parenchymal kidney disease without an alternative primary renal diagnosis. In all cases, chronicity of kidney involvement was required (over three months or longer), and records were reviewed to exclude competing non-DKD etiologies.

Exclusion criteria were suspected/confirmed non-DKD (e.g., glomerulonephritis, polycystic kidney disease, obstructive uropathy, autoimmune renal disease, or active urinary sediment prompting evaluation), acute kidney injury (AKI) within 30 days before or after index, maintenance dialysis, kidney transplantation, pregnancy, or insufficient documentation to ascertain exposure status or outcomes.

Exposure definition

The exposure group comprised patients receiving concurrent empagliflozin and telmisartan. The index date was defined as the first calendar date on which empagliflozin and telmisartan were concurrently active (overlap start) in the medical record. Exposure status was assigned at index (time zero) to avoid immortal time. Persistence of combination therapy was not required for cohort entry in the primary intention-to-treat analysis; instead, treatment persistence was evaluated in sensitivity analyses, including (i) restriction to patients with ≥30 consecutive days of overlap and (ii) an as-treated approach censoring follow-up at discontinuation (coverage gap >60 days) or crossover.

The comparator group included eligible non-albuminuric DKD patients who were receiving telmisartan without any SGLT2 inhibitor therapy at index. For comparators, the index date was defined as the first date within the eligibility window when (a) telmisartan was active, (b) baseline criteria were met, and (c) baseline laboratory measurements were available (aligned to the same baseline window rules as the exposed group). Starting doses and subsequent titrations were recorded when available.

The primary analytic framework was intention-to-treat. Sensitivity analyses applied an as-treated approach, censoring follow-up at discontinuation (coverage gap >60 days) or crossover to SGLT2 inhibitor therapy (comparator)/loss of overlap (exposed).

Outcomes

Primary Outcome

eGFR slope (mL/min/1.73 m^2^/year) estimated from repeated eGFR values from baseline through follow-up. Laboratory-reported eGFR was used; when only serum creatinine was available, eGFR was calculated using the institution’s routine creatinine-based approach as documented in the laboratory system. Creatinine/eGFR values occurring during AKI episodes (per clinical documentation and/or abrupt creatinine rise consistent with AKI) were excluded from slope estimation.

Secondary Renal Outcomes

Secondary renal outcomes included the absolute eGFR change at six and 12 months (±60-day windows). Renal progression was defined as a composite time-to-event outcome comprising a sustained decline in eGFR of greater than or equal to 40% from baseline confirmed over four weeks or later, chronic dialysis, kidney transplantation, or an eGFR of less than 15 mL/min/1.73 m^2^. Incident albuminuria was defined as a urine albumin-to-creatinine ratio (UACR) ≥30 mg/g confirmed on two tests at least four weeks apart among those non-albuminuric at baseline.

Covariates

Prespecified baseline covariates included age, sex, baseline eGFR, baseline UACR, diabetes duration (if available), HbA1c, systolic/diastolic blood pressure, BMI (if recorded), comorbidities (hypertension, coronary artery disease, heart failure, cerebrovascular disease, dyslipidemia), and concomitant medications that may influence kidney outcomes or treatment allocation (e.g., diuretics, nonsteroidal anti-inflammatory drugs (NSAIDs), angiotensin-converting-enzyme (ACE) inhibitors/other ARBs, mineralocorticoid receptor antagonists, glucagon-like peptide-1 (GLP-1) receptor agonists, metformin, insulin, statins). Baseline values were defined using the closest measurement within 90 days pre-index (or on index when recorded on that date).

Statistical analysis

Baseline variables were summarized as mean (standard deviation (SD)) or median (interquartile range (IQR), and n (%). Confounding was addressed using propensity score inverse probability of treatment weighting (IPTW) with stabilized weights; covariate balance was assessed using standardized mean differences (target <0.10). The primary endpoint (eGFR slope) was modeled using weighted linear mixed-effects models with a time × exposure interaction to estimate between-group differences in slope while accounting for within-person repeated measures. Secondary time-to-event outcomes were analyzed using weighted Cox proportional hazards models (hazard ratios (HRs), 95% confidence intervals (CIs)). Missing baseline covariates were handled using complete-case analysis when minimal; otherwise, multiple imputation was applied. Prespecified sensitivity analyses evaluated alternative overlap/as-treated definitions and baseline eGFR strata (α=0.05). Statistical analyses were performed using IBM SPSS Statistics for Windows, Version 30 (Released 2024; IBM Corp., Armonk, New York, United States).

## Results

Study cohort

A total of 120 adults with T2D and non-albuminuric DKD met the eligibility criteria and were included in the analytic cohort. Sixty patients received empagliflozin plus telmisartan (exposed group), and 60 patients received telmisartan without any SGLT2 inhibitor therapy (comparator group).

Baseline Characteristics and Covariate Balance

Baseline characteristics are summarized in Table 1. After propensity score IPTW, the measured baseline covariates achieved acceptable balance (standardized mean difference <0.10), supporting comparability between groups.

**Table 1:** Baseline characteristics and covariate balance Continuous variables are presented as mean ± standard deviation (SD) unless otherwise specified; categorical variables are numbers (percentages). ^†^ Baseline urine albumin-to-creatinine ratio is presented as median (interquartile range). SMD: standardized mean difference; IPTW: inverse probability of treatment weighting; SGLT2: sodium-glucose cotransporter 2

Characteristic	Empagliflozin + telmisartan (n=60)	Telmisartan-based care (no SGLT2 inhibitor) (n=60)	SMD (unweighted)	SMD (IPTW-weighted)
Age, years	59.0 ± 9.5	60.0 ± 9.8	0.10	0.02
Female sex, n (%)	24 (40.0)	23 (38.3)	0.03	0.01
Body mass index, kg/m^2^	27.0 ± 3.8	26.8 ± 3.9	0.05	0.02
Diabetes duration, years	10.0 ± 6.0	10.5 ± 6.5	0.08	0.03
Glycated hemoglobin, %	7.5 ± 0.9	7.6 ± 0.9	0.11	0.04
Systolic blood pressure, mmHg	132 ± 12	133 ± 12	0.08	0.03
Diastolic blood pressure, mmHg	78 ± 8	79 ± 8	0.07	0.03
Baseline estimated glomerular filtration rate, mL/min/1.73 m^2^	77 ± 18	76 ± 18	0.06	0.02
Baseline urine albumin-to-creatinine ratio, mg/g^†^	12 (6-20)	12 (6-21)	0.04	0.02
Hypertension, n (%)	45 (75.0)	46 (76.7)	0.04	0.02
Dyslipidemia, n (%)	38 (63.3)	37 (61.7)	0.03	0.01
Coronary artery disease, n (%)	12 (20.0)	13 (21.7)	0.04	0.02
Heart failure, n (%)	6 (10.0)	7 (11.7)	0.05	0.02
Cerebrovascular disease, n (%)	5 (8.3)	6 (10.0)	0.06	0.02
Diuretics use, n (%)	18 (30.0)	19 (31.7)	0.04	0.02
Non-steroidal anti-inflammatory drug use, n (%)	6 (10.0)	7 (11.7)	0.05	0.02
Angiotensin-converting enzyme inhibitor/other angiotensin receptor blocker (non-telmisartan), n (%)	6 (10.0)	6 (10.0)	0.00	0.00
Mineralocorticoid receptor antagonist use, n (%)	6 (10.0)	5 (8.3)	0.06	0.02
Glucagon-like peptide-1 receptor agonist use, n (%)	5 (8.3)	4 (6.7)	0.06	0.02
Metformin use, n (%)	42 (70.0)	41 (68.3)	0.04	0.02
Insulin use, n (%)	21 (35.0)	22 (36.7)	0.04	0.02
Statin use, n (%)	44 (73.3)	43 (71.7)	0.04	0.02

Primary outcome: annualized eGFR slope

In the weighted longitudinal analysis, empagliflozin plus telmisartan was associated with a slower annual decline in kidney function than telmisartan alone. The estimated mean annualized eGFR slope was -1.15 mL/min/1.73 m^2^/year in the exposed group versus -2.18 mL/min/1.73 m^2^/year in the comparator group, corresponding to a between-group attenuation of 1.03 mL/min/1.73 m^2^/year (Table 2).

**Table 2:** Primary endpoint: annualized eGFR slope Negative values denote a decline in eGFR over time. eGFR: estimated glomerular filtration rate; SGLT2: sodium-glucose cotransporter 2

Analysis set	Empagliflozin + telmisartan (mL/min/1.73 m^2^/year)	Telmisartan-based care (no SGLT2 inhibitor) (mL/min/1.73 m^2^/year)	Between-group attenuation (mL/min/1.73 m^2^/year)
Overall cohort	-1.15	-2.18	1.03

Secondary outcomes

Absolute eGFR change at six and 12 months showed numerically smaller declines in the empagliflozin plus telmisartan group, although between-group differences were not statistically significant (Table 3). Renal progression composite events occurred in three patients (5.0%) in the exposed group and six patients (10.0%) in the comparator group. Incident albuminuria was observed in five patients (8.3%) versus eight patients (13.3%), respectively (Table 3).

**Table 3:** Secondary renal outcomes through 12 months Continuous variables are presented as mean ± SD; categorical variables are numbers (percentages). Six- and 12-month absolute eGFR changes were assessed within prespecified visit windows and compared between groups using IPTW-weighted linear regression (reporting mean difference, Δ, with 95% CI). The renal progression composite was analyzed using an IPTW-weighted Cox proportional hazards model (reporting HR with 95% CI). Incident albuminuria was analyzed using an IPTW-weighted logistic regression model (reporting OR with 95% CI. Renal progression composite is defined as ≥40% sustained decline in eGFR from baseline confirmed over four weeks or later, chronic dialysis, kidney transplantation, or eGFR <15 mL/min/1.73 m^2^. Incident albuminuria is defined as a urine albumin-to-creatinine ratio ≥30 mg/g confirmed on two tests over four weeks apart. CI: confidence interval; eGFR: estimated glomerular filtration rate; HR: hazard ratio; IPTW: inverse probability of treatment weighting; OR: odds ratio; SD: standard deviation; SGLT2: sodium-glucose cotransporter 2

Outcome	Empagliflozin + telmisartan (n=60)	Telmisartan-based care (no SGLT2 inhibitor) (n=60)	Effect estimate (95% CI)	P-value
Absolute eGFR change at six months (±60 days), mL/min/1.73 m^2^	-0.6 ± 4.8	-1.2 ± 5.0	Δ 0.6 (-1.1 to 2.3)	0.48
Absolute eGFR change at 12 months (±60 days), mL/min/1.73 m^2^	-1.1 ± 5.2	-2.2 ± 5.4	Δ 1.1 (-0.9 to 3.1)	0.28
Renal progression composite, n (%)	3 (5.0)	6 (10.0)	HR 0.50 (0.12-2.06)	0.33
Incident albuminuria, n (%)	5 (8.3)	8 (13.3)	OR 0.59 (0.18-1.88)	0.37

## Discussion

In this retrospective cohort of adults with T2D and non-albuminuric DKD (UACR <30 mg/g on serial testing), adding empagliflozin to telmisartan was associated with a meaningfully slower longitudinal loss of kidney function compared with telmisartan-based care without an SGLT2 inhibitor. Because the study was designed around telmisartan-treated patients to test the incremental effect of adding empagliflozin, a sufficiently sized and clinically comparable SGLT2 inhibitor-only subgroup was not available for a robust adjusted analysis. In weighted repeated-measures modeling, the annualized eGFR slope was -1.15 mL/min/1.73 m^2^/year in the combination group versus -2.18 mL/min/1.73 m^2^/year in comparators, yielding a between-group attenuation of 1.03 mL/min/1.73 m^2^/year. Short-horizon changes at six and 12 months were directionally consistent but not statistically significant (Δ +0.6 and +1.1 mL/min/1.73 m^2^, respectively), and event-based outcomes were numerically favorable yet imprecise: the renal progression composite occurred in 3/60 (5.0%) versus 6/60 (10.0%) (HR 0.50, 95% CI 0.12-2.06), while incident albuminuria was observed in 5/60 (8.3%) versus 8/60 (13.3%) (OR 0.59, 95% CI 0.18-1.88). Collectively, the pattern supports the premise that clinically relevant “silent” decline can be modified even before albuminuria emerges, a phenotype increasingly emphasized as biologically active rather than benign [1-3].

The primary finding aligns closely with the limited but highly pertinent real-world evidence base in normoalbuminuric DKD. In particular, Ishibashi et al. reported a slower annual eGFR decline among SGLT2 inhibitor users compared with non-users in non-albuminuric diabetic nephropathy, with effect magnitudes that reinforce the reproducibility of benefit in this phenotype and suggest potential augmentation on a background of RAS inhibition [4]. More broadly, SGLT2 inhibitors have consistently demonstrated kidney-protective signals across clinical trial programs, often in populations enriched for albuminuric risk, with early eGFR “dips,” slower chronic eGFR loss, and reductions in albuminuria progression forming a coherent clinical signature [5,6,12]. Contemporary syntheses similarly position SGLT2 inhibitors and RAS blockade as foundational “pillars,” with combination strategies explicitly framed as a mechanism-guided approach to reduce residual risk in DKD [7,8,13,14].

At the same time, several aspects of our secondary results deviate from what one might expect if the observed slope difference immediately translated into short-term, statistically robust categorical endpoints. The absence of significant differences in six- and 12-month absolute eGFR changes, and the wide CIs around the renal progression composite, is plausibly explained by limited event counts, modest absolute changes over one year, and measurement variability inherent to routine-care creatinine/eGFR capture. Additionally, telmisartan-only care is itself nephroprotective, which compresses between-group separation and makes short-term superiority harder to demonstrate unless the sample is large and follow-up is extended [1,8]. For albuminuria transitions, the numerically lower incident albuminuria rate with combination therapy is directionally consistent with albuminuria “class shift” effects reported with empagliflozin in broader diabetes populations [12], yet the normoalbuminuric baseline state inherently constrains the detectable range of UACR lowering and emphasizes progression prevention rather than regression.

Our findings should also be interpreted in the context of heterogeneous preclinical evidence, which helps explain why the magnitude and visibility of benefit may differ across settings. Some experimental work has shown robust antifibrotic and functional protection with empagliflozin in non-diabetic CKD models, sometimes comparable to telmisartan, and supported by transcriptomic signatures implicating endothelial function and oxidative stress pathways [9,10]. Conversely, in an endothelial nitric oxide synthase (eNOS)-knockout diabetic model where glycaemia was intentionally matched, empagliflozin did not ameliorate albuminuria or histologic injury while telmisartan did, highlighting context dependence and suggesting that metabolic milieu, vascular biology, and model-specific drivers can dominate the treatment signal [11]. Reviews of non-DKD models similarly emphasize that SGLT2 inhibitor effects are not uniform, neutral findings occur, and the balance between hemodynamic versus pleiotropic mechanisms likely varies by disease mechanism and strain [15]. Even combination paradigms show nuance: dapagliflozin increased albuminuria in one 5/6 nephrectomy model, while adding eplerenone mitigated that increase, implying that pathway interactions can reshape renal readouts in ways that may not mirror human DKD phenotypes [16].

Mechanistically, the observed attenuation of eGFR loss with empagliflozin-telmisartan is biologically plausible because the two drug classes target distinct but convergent determinants of progressive nephron injury. RAS blockade reduces efferent arteriolar tone and angiotensin II-mediated profibrotic signaling, thereby lowering intraglomerular pressure and dampening transforming growth factor-β (TGF-β)-linked remodeling [1,17]. SGLT2 inhibition reduces proximal tubular sodium-glucose reabsorption, promoting natriuresis and altering tubuloglomerular feedback, which can decrease hyperfiltration stress and deliver a slower chronic eGFR decline signature that extends beyond glucose lowering [5-7]. Beyond hemodynamics, multiple experimental datasets support anti-inflammatory, antioxidative, and antifibrotic effects of empagliflozin. Reductions in oxidative stress markers, restoration of antioxidant defenses, and attenuation of profibrotic cytokine signaling have been repeatedly documented in diabetic nephropathy models [18,19], while other models implicate complement-related modulation and fibrosis pathway suppression [10]. These mechanisms are particularly relevant to non-albuminuric DKD, where tubulointerstitial inflammation, endothelial dysfunction, hypoxia, and fibrosis may progress despite preserved UACR, making proximal tubular energy stress and interstitial remodeling credible therapeutic targets [1-3].

From a clinical and research perspective, the key novelty of this work is its phenotype-forward evaluation of combination nephroprotection in non-albuminuric DKD using a longitudinal slope endpoint and IPTW-supported comparability, addressing a subgroup often underrepresented in albuminuria-anchored algorithms [12,13]. The findings support future directions that are both pragmatic and mechanistic: larger, multicenter cohorts and prospective studies in normoalbuminuric DKD with longer follow-up to improve event precision; explicit modeling of the early eGFR dip versus chronic slope; exploration of effect modification by baseline eGFR strata, blood pressure control, and metabolic control; and incorporation of biomarkers aligned to oxidative stress, inflammation, tubular injury, and hypoxia pathways suggested by translational syntheses [2,7]. Precision-style strategies may also be valuable, given demonstrated inter-individual variability in response to telmisartan versus empagliflozin versus other agents in albuminuria-focused crossover work [20]. The principal limitations are inherent to the design: retrospective single-center data with potential residual confounding despite weighting; possible exposure misclassification and imperfect adherence ascertainment; limited power for categorical endpoints due to few events; variability in real-world laboratory timing; and restricted generalizability beyond similar care settings and eligibility constraints. In addition, because the cohort was constructed to evaluate the incremental effect of adding empagliflozin on a telmisartan (RAS-blockade) background, a sufficiently sized and clinically comparable subgroup receiving SGLT2 inhibitor therapy without concurrent ACEi/ARB was not available for a robust adjusted “SGLT2 inhibitor-alone” comparison. Nonetheless, the coherent directionality across slope and secondary outcomes, coupled with strong mechanistic plausibility, argues that early combination therapy may be a rational strategy to slow “silent” progression in non-albuminuric DKD, an area where phenotype-specific evidence is still emerging [1,4].

## Conclusions

In this single-centre retrospective cohort of adults with T2D and non-albuminuric DKD, adding empagliflozin to telmisartan was associated with a slower longitudinal decline in kidney function compared with telmisartan-based care alone after adjustment for baseline differences. These findings reflect the incremental effect estimate of SGLT2 inhibition when used on a background of ARB therapy and do not directly evaluate renoprotection with SGLT2 inhibitor monotherapy; however, broader trial programs have demonstrated a coherent kidney-protective signal for SGLT2 inhibitors that is generally consistent with and without background RAS blockade. Short-term kidney function changes and event-based renal outcomes trended in the same direction but were not definitive, likely reflecting limited power and routine-care measurement variability. Overall, the findings support the concept that “silent” progression in non-albuminuric DKD may be modifiable with early combination therapy targeting complementary hemodynamic and pleiotropic pathways, while underscoring the need for larger, multicentre prospective studies with longer follow-up and biomarker-informed phenotyping to confirm durability, clarify treatment-effect heterogeneity, and strengthen clinical applicability.
